# The fourth species of *Sinamma* Lin & Li, 2014 (Araneae, Tetrablemmidae) from China

**DOI:** 10.3897/BDJ.12.e134334

**Published:** 2024-09-16

**Authors:** Ailan He, Jinheng Zhu, Fang Chen, Jing Guo

**Affiliations:** 1 Shaoguan University, Shaoguan, China Shaoguan University Shaoguan China; 2 Administrative Commission of Danxiashan National Park, Shaoguan, China Administrative Commission of Danxiashan National Park Shaoguan China

**Keywords:** taxonomy, haplogyne spiders, new species, description

## Abstract

**Background:**

*Sinamma* Lin & Li, 2014 is a small tetrablemmid genus distributed in China, with three currently known species: *Sinammaoxycera* Lin & Li, 2014 from Guangxi Zhuang Autonomous Region and *S.quadrata* Tong & Li, 2022 and *S.yingae* Tong & Li, 2022 from Guangdong Province.

**New information:**

While examining spider specimens obtained by sifting leaf litter from Danxiashan National Nature Reserve in Guangdong Province of China, we discovered a new species of the genus *Sinamma*, *S.danxia* **sp. nov.**, which is described here.

## Introduction

Members of Tetrablemmidae O. Pickard-Cambridge, 1873, commonly known as tetrablemmid armoured spiders, are very small (< 2 mm length) haplogyne spiders, which can easily be recognised by their strap-like abdominal scuta arranged in parallel (Brescovit and Cizauskas 2019). The family currently comprises 151 species in 27 genera, distributed in southern Asia, Africa and Central and South America (WSC 2024), where they are found in leaf litter, soil and in caves (Burger et al. 2010). Lin and Li (2014) established the genus *Sinamma* Lin & Li, 2014 based on the type species *S.oxycera* Lin & Li, 2014 from Guangxi, China and transferred *Shearellasanya* Lin & Li, 2010 to it, based on some less diagnostic characteristics, such as the pointed eye tubercle, the moderately modified male leg I and the subjectively perceived narrow postgenital plate in the female. Subsequently, Sankaran and Sebastian (2016) transferred *Sinammasanya* (Lin and Li 2010) back to *Shearella*. Later, Cheng et al. (2022) described two more *Sinamma* species (*S.quadrata* Tong & Li, 2022 and *S.yingae* Tong & Li, 2022) from Guangdong, China. To date, three species of this genus have been reported, all distributed in southern China.

In this paper, the fourth species of the genus, *Sinamma* from south China, is described as *S.danxia* sp. nov.

## Materials and methods

All measurements are given in millimetres (mm). Leg measurements are shown as total length (femur, patella, tibia, metatarsus, tarsus). Vulva was cleared in pancreatin solution (Álvarez-Padilla and Hormiga 2007). All specimens are preserved in 75% alcohol. Photographs were taken using an Olympus BX53 microscope equipped with a Kuy Nice CCD camera and were imported into Helicon Focus v. 7 for stacking. Final figures were retouched by the Adobe Photoshop © 2020. The type specimens of the new species are deposited in the School of Biology and Agriculture, Shaoguan University, Shaoguan, China.

Terms and abbreviations used in the text and figures follow Cheng et al. (2022).

## Taxon treatments

### Sinamma danxia

He, Zhu, Chen & Guo sp. nov.

12109A32-F9AD-5E80-B4BE-5E1C917261DE

7ADF9478-8322-46BB-A4BC-3B72039842DA

#### Materials

- **Type status:** Holotype. **Occurrence:** recordedBy: Ailan He; Yingxiang Qiu; Jing Guo; Lijuan Gu; Xiaofei Zhong; individualCount: 1; sex: male; lifeStage: adult; occurrenceID: 73DFDD29-0D7F-5D64-AD59-9D198D1970BC; **Taxon:** order: Araneae; family: Tetrablemmidae; genus: Sinamma; **Location:** country: China; stateProvince: Guangdong; county: Renhua; verbatimLocality: Danxiashan National Nature Reserve (namely Danxia Mountain); verbatimElevation: 201 m; verbatimLatitude: 25.365556°N; verbatimLongitude: 113.739997°E; **Event:** samplingProtocol: Collected by hand picking and sieving leaf litter; year: 2022; month: 12; day: 8; **Record Level:** institutionCode: SGU-Tet001
- **Type status:** Paratype. **Occurrence:** recordedBy: Ailan He; Yingxiang Qiu; Jing Guo; Lijuan Gu; Xiaofei Zhong; individualCount: 18; sex: 5 males, 13 females; lifeStage: adult; occurrenceID: 107F52F4-C778-5F0C-A0BE-D8F319771CCC; **Taxon:** order: Araneae; family: Tetrablemmidae; genus: Sinamma; **Location:** country: China; stateProvince: Guangdong; county: Renhua; verbatimLocality: Danxiashan National Nature Reserve (namely Danxia Mountain); verbatimElevation: 201 m; verbatimLatitude: 25.365556°N; verbatimLongitude: 113.739997°E; **Event:** samplingProtocol: Collected by hand picking and sieving leaf litter; year: 2022; month: 12; day: 8; **Record Level:** institutionCode: SGU-Tet002-019

#### Description

**Male** (holotype). Colouration: body reddish-brown; legs yellowish-brown. Measurements: total length 1.61; carapace 0.75 long, 0.63 wide, 0.49 high; abdomen 1.01 long, 0.76 wide, 0.72 high; clypeus 0.36 high; sternum 0.42 long, 0.44 wide. Length of legs: I 1.90 (0.65, 0.22, 0.41, 0.29, 0.33); II 1.72 (0.54, 0.20, 0.39, 0.27, 0.32); III 1.52 (0.46, 0.18, 0.33, 0.27, 0.28); IV 2.01 (0.60, 0.17, 0.53, 0.37, 0.34). Leg formula: 4123.

**Figure 1.**
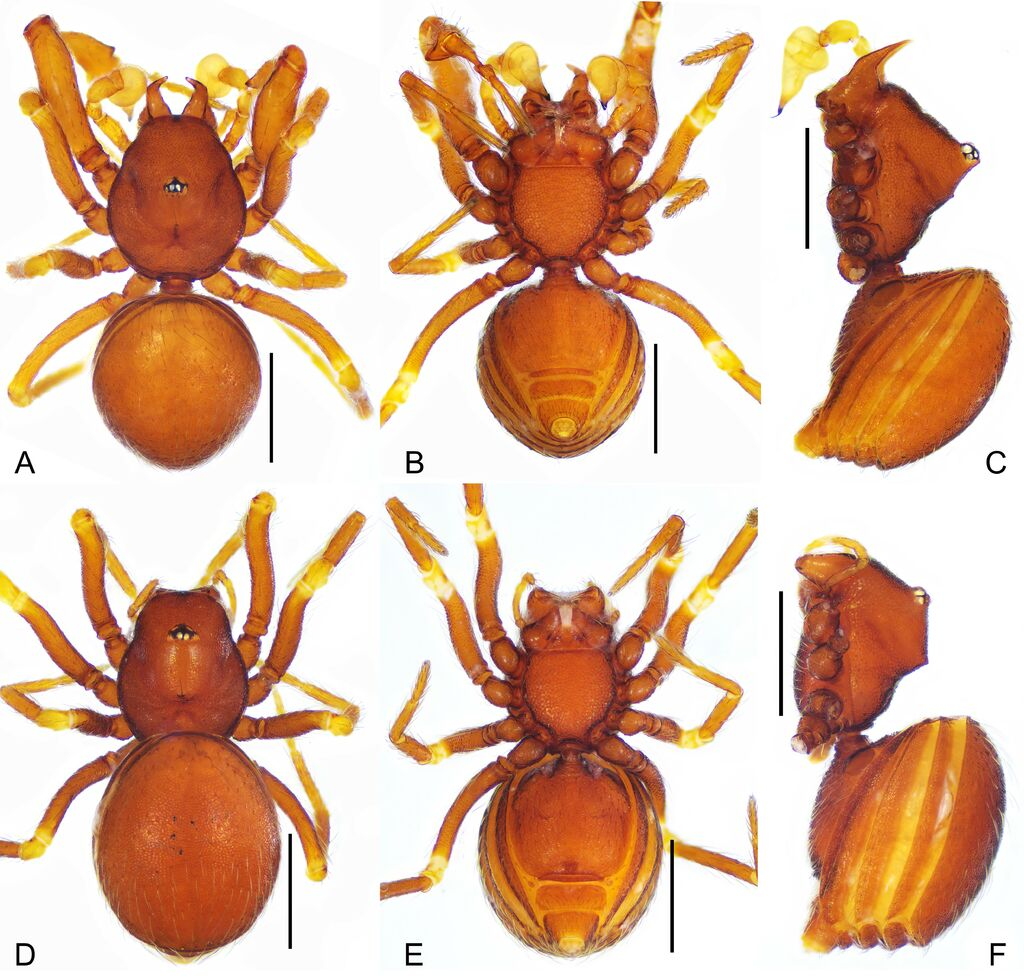
*Sinammadanxia* sp. nov., holotype male (A–C), paratype female (D–F). **A, D** Habitus, dorsal view; **B, E** Habitus, ventral view; **C, F** Habitus, lateral view. Scale bars: 0.5 mm (A–F).

**Figure 2.**
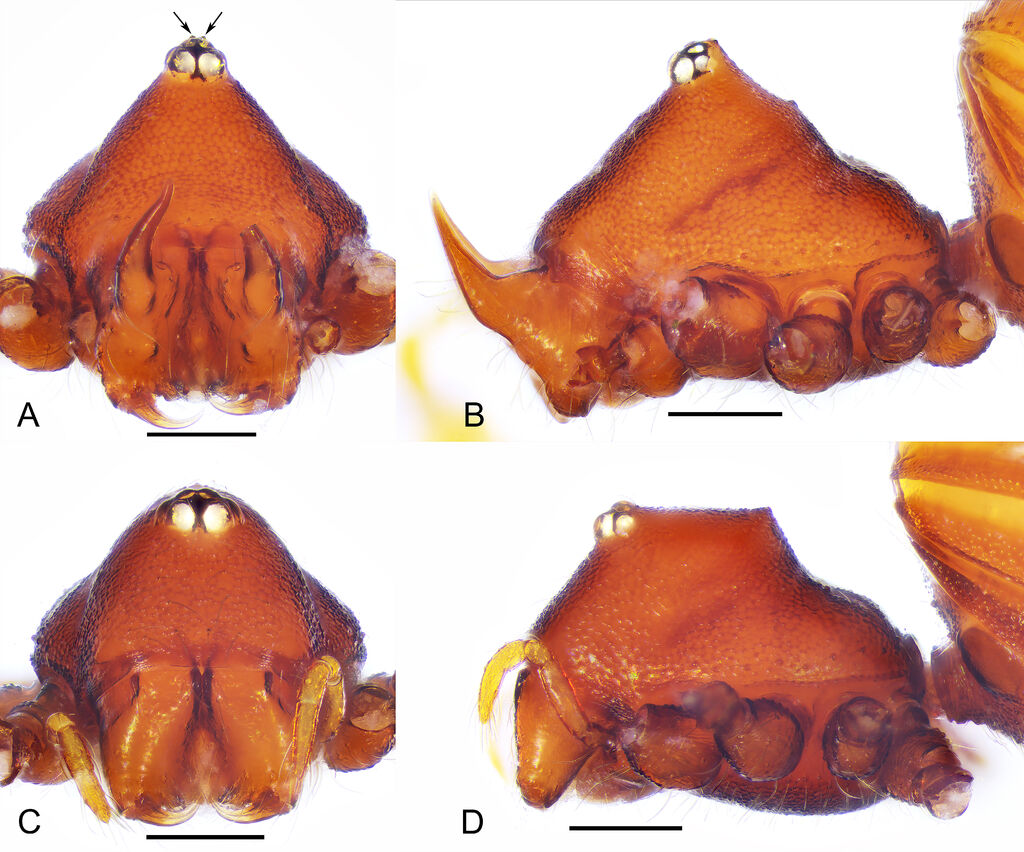
*Sinammadanxia* sp. nov., holotype male (A–B), paratype female (C–D). **A, C.** Prosoma, anterior views; **B, D.** Prosoma, lateral view. Scale bars: 0.2 mm (A–D). The arrows indicate small tubercles in the ocular area.

**Figure 3.**
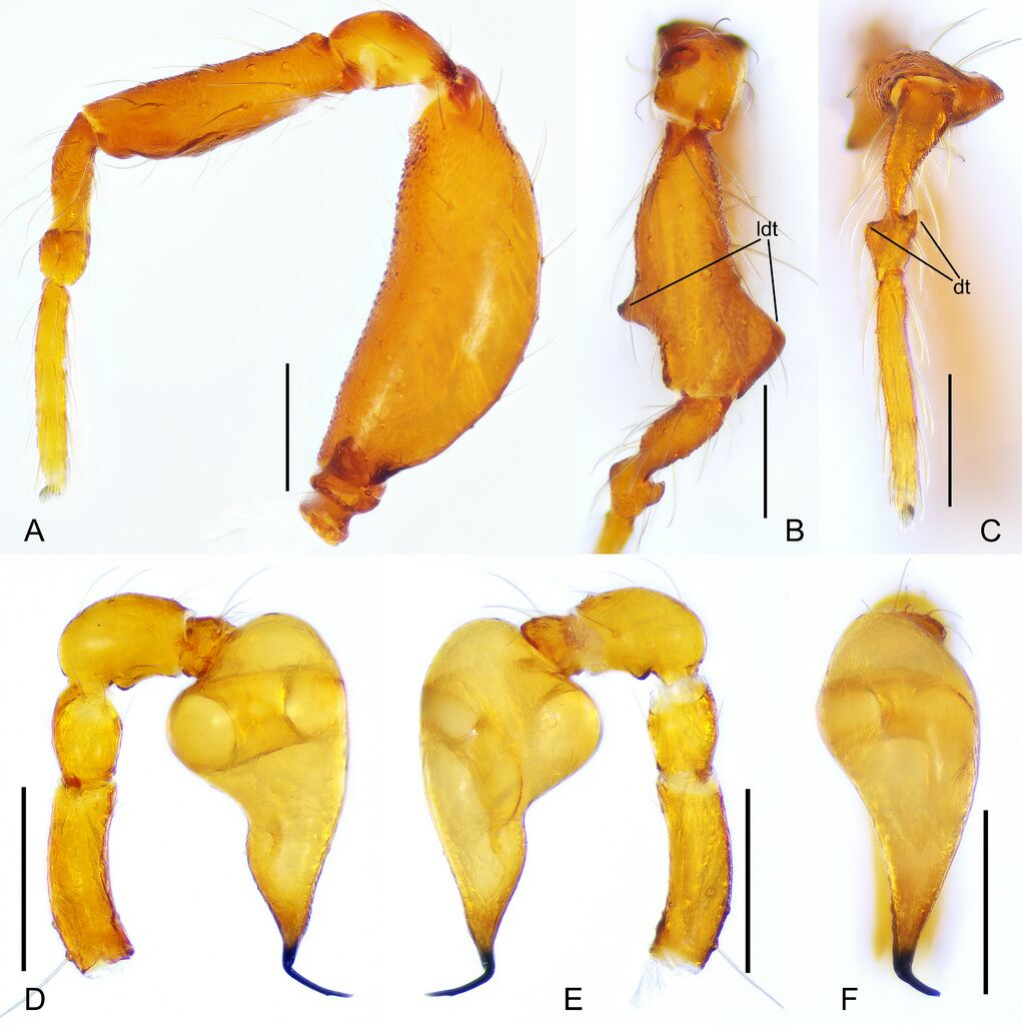
*Sinammadanxia* sp. nov., holotype male. **A** Left leg I, retrolateral view; **B** Left tibia I, dorsal view; **C** Left metatarsus I and tarsus I, dorsal view; **D** Left palp, prolateral view; **E** Same, retrolateral view; **F** Same, dorsal view. Abbreviations: dt = distal tubercles; ldt = large distal tubercles. Scale bars: 0.2 mm (A–F).

Carapace (Fig. 1A, C, Fig. 2A and B): reticulated, margin with small denticles; ocular area distinctly raised (Fig. 2A and B), with a pair of very small tubercles behind and almost same height as ocular area; clypeus very high, anterior margin rounded (Fig. 2A); cheliceral horns long, basally wide, distally crooked, with tips parallel and pointing antereodorsally (Fig. 2B); sternum with sparse setae, reticulate (Fig. 1B). Legs: femur I swollen (Fig. 3A); tibiae I–III with 3 trichobothria, respectively, tibia IV with 4 and metatarsi I-IV with one; tibia I with two large triangular subdistal tubercles, retrolateral one larger and prolateral one smaller (Fig. 3B); metatarsus I constricted subdistally and with two small tubercles (Fig. 3C).

Abdomen (Fig. 1A–C): booklung covers oval, reddish-brown; dorsal scutum oval, finely reticulated; ventral scutum reticulated; postgenital plate smooth, narrow, slightly shorter in width to pre-anal plate; pre-anal plate slightly curved.

Palp (Fig. 3D–F): femur smooth, slightly curved at mid-ventral side; patella approximately 1/2 of femur in length, connected to tibia sub-basally; bulb long, pyriform, with a distinct contraction in middle of ventral surface; embolus long, spiniform, strongly sclerotised and bending at a nearly right angle at base towards dorsally; sperm duct extending, visible through the bulbal integument.

**Female** (paratype). Colouration: same as in male. Measurements: total length 1.60; carapace 0.69 long, 0.57 wide, 0.37 high; abdomen 1.02 long, 0.81 wide, 0.76 high; clypeus 0.22 high; sternum 0.40 long, 0.41 wide. Length of legs: I 1.72 (0.55, 0.18, 0.40, 0.26, 0.33); II 1.61 (0.49, 0.18, 0.36, 0.26, 0.32); III 1.47 (0.43, 0.18, 0.31, 0.27, 0.28); IV 1.92 (0.58, 0.16, 0.50, 0.36, 0.32). Leg formula: 4123.

Carapace (Fig. 1D, F, C and D): cephalic part raised, dorsal edge straight in lateral view (Fig. 2D), ocular area not raised, clypeus lower than in male; cheliceral horn absent. Legs as in male, except for leg I not swollen.

Abdomen (Fig. 1D–F): ventral episgastric scutum reticulated; postgenital plate straight, slightly shorter in width to pre-anal plate; pre-anal plate rectangular-shape; perigenital plate small, oval.

**Figure 4.**
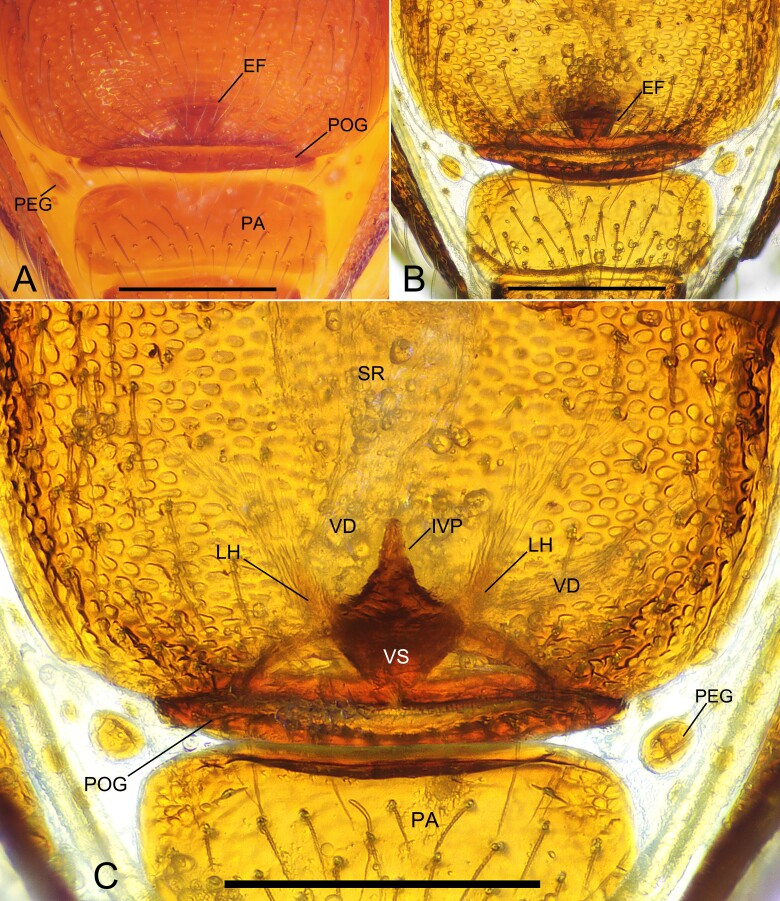
*Sinammadanxia* sp. nov., paratype female. **A** Genital area, ventral view; **B** Genital area, ventral view (cleared in pancreatin solution); **C** Endogyne, dorsal view. Abbreviations: EF = epigynal fold; IVP = inner vulval plate; LH = lateral horn; PA= pre-anal plate; PEG = perigenital plate; POG = postgenital plate; SR = seminal receptaculum; VD = vulval duct; VS = vulval stem. Scale bars: 0.2 mm (A–C).

Genitalia (Fig. 4A–C): epigynal fold wide, arched (Fig. 4A and B); vulval stem wide, inverted triangular, strongly sclerotised; lateral horns slightly sclerotised, supporting the base of membranous vulval ducts that connect spermathecae; inner vulval plate triangle-shaped, rugose, slightly sclerotised; spermathecae translucent, oval, membranous (Fig. 4C).

#### Diagnosis

Amongst the congeners, the new species can be easily distinguished from *S.oxycera* and *S.quadrata* by the presence of pointed cephalic tubercles in the latter two species (both sexes in *S.oxycera* and males in *S.quadrata*). The new species is similar to *S.yingae* Tong & Li, 2022 in that both males and females lack pointed cephalic tubercles and in the pattern of leg tubercles on tibia and metatarsus I, but can be recognised by: Cheng et al. (2022); 1) dorsal surface of the cephalic part in females straight in lateral view (D) (vs. cephalic part slightly sloping (Cheng et al. (2022): fig. 5F)); 2) metatarsus I without proximal tubercle (Fig. 3B and C) (vs. metatarsus I with one proximal tubercle (Cheng et al. (2022): fig. 3D)); 3) the length of the male palpal patella about 1/2 of the femur in length (Fig. 3D and E) (vs. the patella about 2/3 femur in length (Cheng et al. (2022): figs. 6A and B)); 4) embolus bending at a nearly right angle at base (Fig. 3D and E) (vs. the embolus slightly curved (Cheng et al. (2022): figs. 6A and B)); 5) vulval stem inverted triangular and inner vulval plate triangular with rugose surface (Fig. 4C) (vs. vulval stem inverted trapezoidal and inner vulval plate finger-like with smooth surface (Cheng et al. (2022): fig. 7C)).

#### Etymology

The specific epithet refers to the type locality; noun in apposition.

#### Distribution

**Figure 5.**
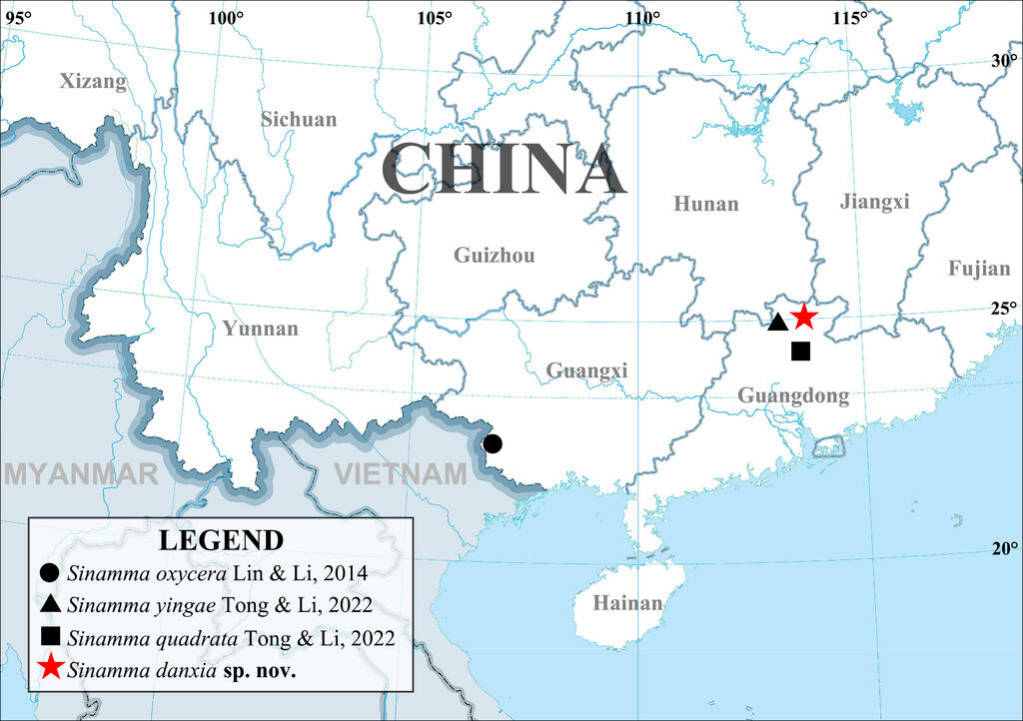
Map showing the currently known localities of *Sinamma* spp.

Known only from the type locality (Guangdong, China) (Fig. 5)

## Identification Keys

### Key to known species of *Sinamma*

**Table:** 

1	Males	2
–	Females	5
2	With pointed eye tubercles (Lin and Li (2014): figs. 1E and G; Cheng et al. (2022): figs. 1G and H)	3
–	Without pointed eye tubercles (Cheng et al. (2022): figs. 5G and H; Figs. 2Fig. 2A and B)	4
3	Palpal bulb quadrangular, embolus belt-shaped (Cheng et al. (2022): figs. 2A and B)	*S.quadrata*
–	Palpal bulb pyriform, embolus thread-like (Lin and Li (2014): figs. 2A and B)	*S.oxycera*
4	Cheliceral horn short, with the tips pointing towards each other (Cheng et al. (2022): figs. 5G and H)	*S.yingae*
–	C heliceral horn long, with their tips parallel and pointing towards dorsum	*S.danxia* sp. nov.
5	Carapace with a pair of cephalic tubercles (Lin and Li (2014): figs. 1F and H)	*S.oxycera*
–	Carapace without cephalic tubercles (Cheng et al. (2022): figs. 1I, 5I; C and D)	6
6	Cephalic part slightly elevated, the highest point of carapace at posterior 2/3 (Cheng et al. (2022): fig. 1F); posterior part of sternum strongly bulged (Cheng et al. (2022): fig. 1F)	*S.quadrata*
–	Cephalic part not elevated, the highest point of carapace at ocular area (Cheng et al. (2022): figs. 5F and D); posterior part of sternum flat (Cheng et al. (2022): figs. 5F and D)	7
7	Cephalic part slightly sloping (Cheng et al. (2022): fig. 5F); vulval stem inverted trapezoidal (Cheng et al. (2022): fig. 7C)	*S.yingae*
–	Cephalic part extremely flat (D); vulval stem inverted triangular (Fig. 4C)	*S.danxia* sp. nov.

## Supplementary Material

XML Treatment for Sinamma danxia
